# The Mediterranean diet and periodontitis: A systematic review and meta-analysis

**DOI:** 10.1016/j.heliyon.2024.e35633

**Published:** 2024-08-05

**Authors:** Yasmina Aalizadeh, Nima Khamisi, Parastoo Asghari, Amirhossein Safari, Mahtab Mottaghi, Mohamad Hosein Taherkhani, Anahita Alemi, Masoume Ghaderi, Mohammad Rahmanian

**Affiliations:** a Department of Pediatric Dentistry, School of Dentistry, Islamic Azad University (khorasgan Branch), IAU (Khorasgan Branch), University Blvd, Jey St, Arqavanieh, Isfahan, 81551-39998, Iran; b School of Dentistry, Islamic Azad University (Khorasgan Branch), IAU (Khorasgan Branch), University Blvd, Jey St, Arqavanieh, Isfahan, 81551-39998, Iran; c Department of Nutrition, Faculty of Medicine, Mashhad University of Medical Sciences, Knowledge and Health City, At the End of Shahid Fakouri Blvd (In Front of Fakouri 94), Mashhad, 99191-91778, Iran; d School of Dentistry, Tehran Islamic Azad University of Medical Sciences, No. 4 9th Neystan Pasdaran St, Tehran, 1946853314, Iran; e School of Dentistry, Mashhad University of Medical Sciences, At the Beginning of Vakil Abad Blvd., in Front of Mellat Park, Mashhad, 9177948959, Iran; f School of Dentistry, Tehran University of Medical Sciences, TUMS, North Kargar Ave, Amirabad, Tehran, 1439955934, Iran; g Student Research Committee, School of Dentistry, Zanjan University of Medical Sciences, Dr.Sobouti Blvd, Zanjan, 4513956184, Iran; h Student Research Committee, School of Medicine, Shahid Beheshti University of Medical Sciences, SBUMS, Arabi Ave, Daneshjoo Blvd, Velenjak, Tehran, 1983963113, Iran

**Keywords:** Mediterranean diet, Periodontitis, Nutrition, Oral health

## Abstract

Periodontitis is a severe oral health condition that affects the soft tissue and bone supporting the teeth. The Mediterranean diet has been proposed as a potential contributor to reducing the risk of periodontitis. This systematic review and meta-analysis aims to explore the association between adherence to the Mediterranean diet and periodontitis. A comprehensive literature search from 1992 to January 2024 was conducted across multiple databases, including PubMed, Scopus, Web of Science, and Google Scholar. The included studies were clinical trials, randomized controlled trials, and observational studies that evaluated the impact of the Mediterranean diet on periodontitis. Data extraction and quality assessment of the included studies were performed using standardized protocols. A meta-analysis was conducted to combine effect sizes from multiple studies. This review included seven studies, comprising one cohort study, five cross-sectional studies, and one randomized controlled trial. While some studies reported a potential link between Mediterranean diet adherence and periodontitis, the overall analysis did not demonstrate a significant association. The meta-analysis revealed an Odds Ratio (95 % Confidence Interval) of 0.77 (0.58, 1.03) for the association between adherence to the Mediterranean diet and periodontitis *(p* = *0.08)*. This systematic review and meta-analysis found no statistically significant association between periodontitis and Mediterranean diet adherence. Future research should prioritize the implementation of rigorous clinical studies with longer follow-up periods to better understand the causal association between the Mediterranean diet and periodontitis. Observational studies with larger sample sizes are needed to establish more conclusive evidence regarding the impact of dietary patterns on periodontal health.

## 1 Introduction

Periodontitis is a common oral inflammatory disease [1]. Studies indicate that 42 % of people in the US have periodontitis, with severe instances accounting for 7 %–11 % of cases [2,3]. This condition destroys the teeth-supported tissues, resulting in tooth loss and lowering the patient's quality of life. Furthermore, it has been related to the severity of systemic diseases [1], specifically diabetes mellitus and cardiovascular disease [3].

Previous studies have identified various risk factors related to periodontitis. For instance, a systematic review by Kim et al. demonstrated a positive relationship between obesity and periodontitis [4]. Additionally, Dental plaque accumulation and poor oral hygiene are widely recognized as significant risk factors for the beginning and progression of periodontitis [5]. Subgingival calculus was associated with the onset of periodontitis, whereas dental plaque causes its ongoing progression [6]. There are surgical and nonsurgical treatments for periodontitis. Nonsurgical therapies include root planning and scaling, whereas surgical therapies include regenerative and corresponding operations [2]. However, It has been shown that surgical therapy may result in postoperative and long-term complications such as infection, discomfort, pain, bleeding, and tooth sensitivity [7]. Primary prevention stands as the cornerstone of disease management, offering efficient and cost-effective strategies. This approach includes optimizing dental hygiene and professional teeth cleaning [8], reducing modifiable risk factors, and addressing underlying causes [2]. Lertpimonchai et al.'s systematic review revealed that poor oral hygiene increases the risk of periodontitis by two to five times. However, consistent toothbrushing and regular dental visits could decrease this risk [5]. Unhealthy behaviors such as smoking, alcohol consumption, lack of physical activity, and a diet high in salt, saturated fats, and sugar contribute to periodontal diseases [9].

A nutritious diet reduces the risk of periodontitis and other chronic illnesses [10]. Increasing the consumption of healthy foods high in fiber, grains, and vegetables can protect the periodontal tissue. Conversely, a diet high in processed foods, fat, sugar, and salt can increase the risk of developing systemic and inflammatory disorders, which might contribute to periodontal damage [11]. The Mediterranean diet is a nutritious eating pattern characterized by its low intake of red meat, sodium, sugar, and saturated fat. Instead, it emphasizes foods of plant origin like grains, fish, vegetables, fruits, and nuts [12,13]. Studies have demonstrated the potential of the Mediterranean diet to reduce the incidence and delay the onset of cardiovascular disease [14], breast cancer [15], diabetes [16], and cognitive decline [17].

A limited number of studies have investigated the relationship between dietary habits and periodontitis, including a systematic review and meta-analysis conducted by Sáenz-Ravello et al. [18]. This review included four randomized controlled trials [[19], [20], [21], [22]] involving 136 patients with gingivitis, revealing a non-significant reduction in periodontal pocket depth among those in the intervention group. Several studies have indicated a connection between the prevalence of periodontitis and the Mediterranean diet [9,13,23]. However, some studies found no relationship between the prevalence of periodontitis and the Mediterranean diet [12]. To our knowledge, no systematic review or meta-analysis has been conducted to evaluate the relationship between the Mediterranean diet and periodontitis. Therefore, the present study aims to assess current data on this association and perform a meta-analysis for the first time to explore whether Mediterranean diet adherence could potentially reduce the incidence of periodontitis.

## 2 Materials and methods

### 2.1 Literature search selection criteria, search methods, data collection, and data analysis

The reporting guidelines for this systematic review and meta-analysis follow PRISMA [24]. One reviewer developed a search strategy to investigate the effect of the Mediterranean diet on periodontitis (Y.A.). A wide range of Mesh terms related to the impact of the Mediterranean diet on periodontitis were used in the search strategy; modified keywords were used to search each database. The search query is displayed in Table 1. The search included PubMed, Web of Science, Scopus, and Google Scholar from 1992 to January 2024, covering the duration of the search process.

**Table 1:** Search strategy.

Database	Search Terms	Date of Search
PubMed	((Mediterranean Diet [Title/Abstract]) OR (Diets, Mediterranean [Title/Abstract])) OR (Mediterranean Diets[Title/Abstract]) AND (((Periodontitides[Title/Abstract]) OR (Pericementitis[Title/Abstract])) OR (Pericementitides[Title/Abstract])) OR (Periodontitis[Title/Abstract])	14/1/2024
Scopus	(TITLE-ABS-KEY (periodontitis) OR TITLE-ABS-KEY(periodontitides) OR TITLE-ABS-KEY(pericementitis)) AND (TITLE-ABS-KEY(mediterranean AND diet) OR TITLE-ABS-KEY(diets, AND mediterranean) OR TITLE-ABS-KEY(mediterranean AND diets))	14/1/2024
Web of Science	(((TS=(Periodontitis)) OR TS=(Pericementitis)) OR TS=(Pericementitides)) OR TS=(Periodontitides) AND ((TS= (Mediterranean Diet)) OR TS=(Diets, Mediterranean)) OR TS=(Mediterranean Diets	14/1/2024
Google scholar	All in title: periodontitis and Mediterranean periodontitis AND “Mediterranean diet"	14/1/2024

### 2.2 Study selection and data collection

Two independent team members were involved at each stage, from title screening to data collection, resolving disagreements through discussion (Y.A. and M.M.). Studies were included based on the following PICO/PECO strategy:

Participants (P): Adults aged 18 to 60 who participated in either:

• A Mediterranean diet intervention to treat periodontitis.
• An observational study evaluating adherence to the Mediterranean diet to prevent periodontitis.

Intervention (I)/Exposure (E): Studies were selected based on an intervention group demonstrating high adherence to the Mediterranean diet among patients with periodontitis. The level of adherence was measured by various scoring systems, including:

• Mediterranean Diet Adherence Screener (MEDAS) [25]: Typically, a score ranging from 0 to 4 indicates low adherence, while scores between 5 and 9 signify medium adherence, and scores from 10 to 14 represent high adherence [13].
• Mediterranean Diet Serving Score (MDSS) [26]: The maximum MDSS value is 24 points, with a cut-off of ≥14 points used to indicate compliance with the principles of the Mediterranean diet [27].
• Italian Mediterranean Index score (ItMedIndex) [28]: Higher scores represent better adherence, with specific thresholds varying by study [29].
• Alternate Mediterranean diet score (aMed) [30]: The aMed score, which ranged from 0 to 9, indicated the level of adherence to the Mediterranean diet pattern. Researchers categorized aMed scores into three groups: 0–3 represented low adherence to the Mediterranean diet pattern, 4–6 represented moderate adherence, and 7–9 represented great adherence [31].
• Validated Questionnaires:
• Food Frequency Questionnaire (FFQ): Used to estimate adherence based on frequency and portion size of food intake [32].
• Adherence to Mediterranean Diet Questionnaire (QueMD): Higher scores reflect greater adherence [30].

Periodontitis is classified into three stages: mild, moderate, and severe, according to previous studies. These stages are defined as follows [33,34]:

• Mild Periodontitis: Characterized by having at least two interproximal sites with Clinical Attachment Loss (CAL) of 3 mm and at least two interproximal sites with probing depths of 4 mm (on different teeth) or at least one site with a PD of 5 mm.
• Moderate Periodontitis: Defined by having at least two interproximal sites with clinical CAL of 4 mm or more (on different teeth) or at least two interproximal sites with probing depths of 5 mm or more, also on different teeth.
• Severe Periodontitis: Identified by having at least two interproximal sites with CAL of 6 mm or more (on different teeth) and at least one interproximal site with a probing depth of 5 mm or more.

Comparison (C): A control group:

• Without adherence to the Mediterranean diet.
• With adherence to a different diet.

Outcome (O): Studies that evaluated the following periodontal indices [[35], [36], [37]]:

• Bleeding on Probing (BOP)
• CAL
• Probing Pocket Depth (PPD)
• Gingival Index (GI)
• Plaque Index (PI)
• Periodontal Inflamed Surface Area (PISA)
• other periodontal indices

The following are the exclusion criteria for this study:

1. Non-English studies
2. Animal and laboratory studies
3. Studies that do not explicitly mention the effect on “periodontitis."
4. Studies investigating the impact of healthy diets without specific emphasis on the Mediterranean diet.
5. Review studies.
6. Editorials, opinion articles, theses, and grey literature.

Two reviewers (A.A. and M.R.) performed the data extraction process independently. For each study, a data extraction form was employed to collect relevant data for inclusion in this analysis, as mentioned below:

• Study design
• Patient characteristics (Age, Ethnicity, Education, Income, Obesity, Body mass index (BMI), alcohol consumption, smoking habits, Mediterranean diet index/score, dental visits, brushing frequency, systematic diseases, and periodontitis stage)
• Periodontal variables before and after the intervention
• Summary statistics (means, medians, or percentages)
• Explanation of differences among treatment groups

### 2.3 Assessment of methodological quality

Two independent reviewers employed Joanna Briggs Institute (JBI) critical appraisal tools to assess the quality of our study's included articles, encompassing randomized controlled trials, cross-sectional, and cohort studies (N.K. and Y.A.). A third reviewer settled disagreements during the quality appraisal process (M.R.). Randomization, blinding, and analytical methods were assessed using specific JBI checklists tailored to each study design.

### 2.4 Certainty assessment

The certainty of the included studies was assessed using the Grading of Recommendations Assessment, Development, and Evaluation (GRADE) tool, which classifies the level of evidence into four categories: high, moderate, low, and very low. The GRADE approach considered several critical factors [38]:

• **Risk of Bias:** Evaluating the methodological quality of the included studies.
• **Inconsistency**: Assesses the differences in study results.
• **Indirectness**: Assesses the applicability of the evidence to the population, intervention, comparator, and outcomes of interest.
• **Imprecision**: Reviews the confidence intervals and sample sizes of the included studies.
• **Publication Bias:** Assesses the likelihood of selective publication of studies.

Two reviewers (M.M. and Y.A.) independently assessed each outcome. Disagreements were resolved through discussion or, if necessary, by consulting a third reviewer (M.H.). The results of the certainty assessment are summarized in Table 3.

**Table 2:** The characteristics of the included studies in this systematic review and meta-analysis.

Publication year	author	country	type of study	follow up duration	population	sex(female%)	adjustments	outcomes	quality score
2023	Wu et al. [40]	USA	Cross-sectional	NA	8290	4228(51 %)	sex, age, ethnicity, education, marital status, and poverty to income ratio, smoking status, diabetes, hypertension, total energy intake, and sedentary time, BMI and waist circumference	adherence to Mediterranean diet → periodontitis↓	8/8
2023	Shakeel et al. [42]	India	clinical trial	3 months	150	60(40 %)	NA	adherence Mediterranean diet among patients of diabetes mellitus → periodontits ↓	5/13
2022	Radić et al. [41]	Croatia	Cross-sectional	NA	89	40(44.9 %)	age, sex, transplantation years, eGFR, presence of arterial hypertension, presence of	low adherence to Mediterranean diet in kidny transplant recipients → periodontitis↑	5/8
2022	Marruganti et al. [39]	Italy	cohort	6 months	120	50(31.6 %)	Probing pocket depth at base line, FMPS, FMBS, the number of the mobile teeth, end point therapy, + diabetes + household disposable income + 3 M FMPS	Subjects with low adherence to Mediterranean diet → worst results in the treatment process of periodontitis	7/11
2021	Marruganti et al. [3]	Italy	Cross-sectional	NA	235	136(57.8 %)	age, sex, smoking, and brushing frequency.	low Mediterranean diet adherent + lack of regular exercise → periodontitis stage II and III ↑	8/8
2021	Altun et al. [13]	Germany	Cross-sectional	NA	6209	1814(50.7 %)	age, sex, and physical activity	adherence to Mediterranean diet → periodontal dieases↓	6/8
2020	Iwasaki et al. [12]	Morocco	Cross-sectional	NA	1075	774(72 %)	age, sex, and oral health behaviour	The Mediterranean diet was not significantly associated with periodontitis among young Moroccans.	8/8

**Table 3:** GRADE tool to evaluate the certainty of the evidence.

Certainty assessment							Number of patients	Effect		Certainty	Importance
Number of the studies	Study design	Risk of bias	Inconsistency	Indirectness	Imprecision	Other considerations		Relative(95 % CI)	Absolute(95 % CI)		
3	cross-sectional	serious	Not serious	Not serious	Not serious	none	15574	–	1.77	⨁⨁⨁◯	Important

### 2.5 Statistical analysis

Review Manager, version 5.4 (The Nordic Cochrane Centre, Copenhagen, Denmark) was used for the statistical analysis. The effect sizes were measured using ORs (95 % Confidence Interval). A two-sided p-value of <0.05 was regarded as statistically significant. A generic inverse-variance random effect combined the findings regarding the association between Mediterranean diet adherence and periodontitis. A sensitivity analysis was conducted to evaluate how omitting a single article from the analysis at each iteration could affect the overall pooled findings. The Q-test and I^2^ statistics assessed the between-study heterogeneity; I^2^ statistics of >50 % were judged statistically significant. Publication bias was estimated using Begg's funnel plot and Egger's test.

## 3 Results

**Fig. 1:**
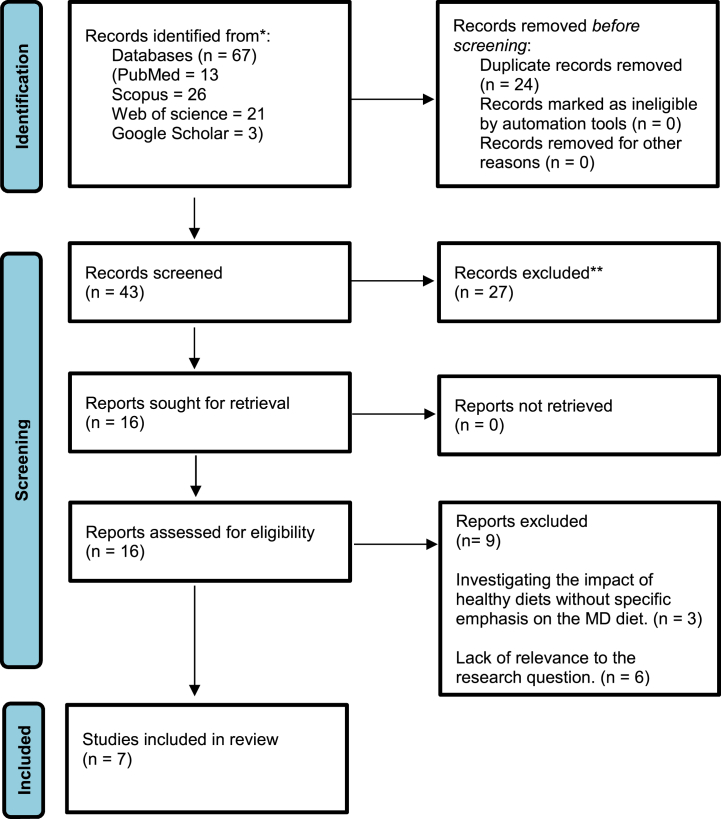
A flow diagram of study selection process to identify the eligible studies.

Fig. 1 depicts the article selection process. One hundred thirty studies were found via database searches. After eliminating duplicates, forty-three articles remained. After excluding twenty-seven studies based on their titles and abstracts, sixteen studies were selected for a thorough review of the full texts. Ultimately, nine articles were excluded: six studies lacked relevance to the research question, and three did not specifically focus on the Mediterranean diet when investigating the impact of healthy diets. Finally, the current review included seven studies categorized as follows: one cohort study [39], five cross-sectional studies [3,12,13,40,41], and one randomized controlled trial [42]. There were 120 cases (total: 120) in the cohort study, 15898 cases (total:15898) in the cross-sectional studies, and 75 cases and 75 controls (total:150) in the randomized controlled trial, with 16168 people overall in all of the studies (16193 cases and 133 controls). One investigation was carried out in Germany [13], two in Italy [3,12,39], one in the USA [40], one in India [42], one in Morocco [12], and one in Croatia [41]. The interventions ranged in length from 6 weeks to 5 years. Other relevant findings from studies are shown in Table 2.

### 3.1 Patient characteristics

The average age of the participants was 43.85. The range of age was between 18 and 80, and the percentage of female patients was 43.92 percent. Further data are shown in Table 2.

### 3.2 The quality of the included studies

Observational study checklists revealed that the included cohort study, Marruganti et al. [39], exhibited minimal bias. The cross-sectional studies showed minimal bias except for Radić et al. [41] and Altun et al. [13], where the inclusion criteria were not explicitly stated. In contrast, the randomized controlled trials exhibited a substantial risk of bias regarding randomization, participant selection, group concealment, treatment administration, and identical treatment (Table 2).

### 3.3 Effect of the Mediterranean diet on periodontitis

Marruganti et al. [39] indicated that smoking and low aMed were negatively correlated with changes in CAL, recession (REC), and PPD. Furthermore, reduced therapeutic endpoint rates were noted in cases with inadequate Mediterranean diet adherence. Radic et al. [41] reported that Kidney transplant recipients (KTRs) who followed the Mediterranean Diet, removed their dental plaque regularly, particularly through plant-based recommendations, and maintained daily teeth cleaning exhibited notable improvements in dental health, especially among those who followed the olive oil suggestions. Regular cereal eating and frequent nut consumption were linked to decreased PPD, interdental CAL, and tooth plaque. The study of Marrugati et al. [3] showed that participants who had poor Mediterranean diet adherence had less favorable biometric periodontal characteristics (i.e., %PPD 5–6 mm, %PPD >4 mm, tooth loss due to periodontal reasons, furcation involvement, numerous bleeding pockets, tooth mobility). A decreased incidence of the third and fourth stages of periodontitis (29.66 %) was strongly linked with increased adherence to the Mediterranean diet, even after controlling for smoking, frequency of brushing, age, and sex. There was a significant rise in the incidence of advanced stages of periodontitis within the subgroup with ‘low Mediterranean diet adherence and low or moderate physical activity’ at 73.68 % and the ‘low adherence to Mediterranean diet but high physical activity’ subgroup at 59.09 %, compared to the ‘high Mediterranean diet adherence and moderate or low physical activity’ subgroup (30.14 %) and a subgroup of ‘high adherence to Mediterranean diet with high physical activity’ (28.89 %). The periodontal status was notably poor in the initial categories compared to the finals. When participants showed poor adherence to the Mediterranean diet, the risk of developing Stage III/IV periodontitis increased nine times for those with low/moderate physical activity levels and ten times for those with high physical activity levels. Furthermore, Body Mass Index (BMI) partially mediates approximately nine percent of the effect of physical activity and the Mediterranean diet on periodontitis in stages III and IV [19].

### 3.4 Statistical focus on the Mediterranean diet

**Fig. 2:**
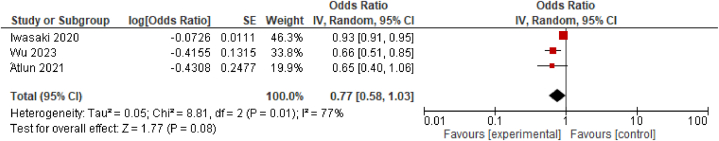
Forrest plot evaluating the association between Mediterranean diet adherence and periodontitis.

Fig. 2 depicts the random-effects forest plot representing the relationship between Mediterranean diet adherence and periodontitis. The pooled Odds Ratio (95 % Confidence Interval) for the association between Mediterranean diet adherence and periodontitis was 0.77 (0.58, 1.03), with a significant degree of heterogeneity (I^2^: 77 %, *p* = *0.01*).

### 3.5 Certainty of evidence assessment

We assessed the certainty of evidence at the outcome level across studies using the GRADE approach [43]. The results are presented in Table 3.

## 4 Discussion

This is the first systematic review and meta-analysis to investigate the relationship between the Mediterranean diet and periodontitis. However, another systematic review by Sáenz-Ravello et al. [18] assessed the effect of healthy dietary patterns on clinical periodontal parameters. Their study included four RCTs [[19], [20], [21], [22]] that evaluated the Mediterranean diet in 136 patients with gingivitis, showing a clinically insignificant reduction in PPD in the intervention groups. Similarly, our meta-analysis found no significant association between adherence to the Mediterranean diet and periodontitis.

Typically, two major dietary patterns are often compared—the Mediterranean diet and the Western diet. The Mediterranean diet focuses on olive oil, fresh seasonal vegetables, cereals, and plants, with limited meat consumption. In contrast, the Western diet is characterized by a high intake of high-fat dairy products, processed foods, and red meat [44]. From a biological point of view, consuming a Western diet leads to low-grade inflammation, contributing to the development of various non-communicable diseases, including periodontitis. In contrast, high adherence to the Mediterranean diet has been associated with a lower prevalence of advanced periodontitis, likely attributable to the combined anti-inflammatory properties of its components [3]. Fish and olive oil, abundant in omega-3 fatty acids, mitigate inflammatory responses. Meanwhile, vegetables, legumes, and fruits provide dietary fiber, vitamins, minerals, and trace elements with anti-inflammatory and antioxidant effects. Whole grains and fruits also show a negative correlation with periodontitis. Conversely, red meat, a source of saturated fatty acids, is associated with inflammation and oxidative stress. Due to its polyphenolic component, moderate red wine consumption may also help prevent periodontitis [40].

Several studies indicated that greater adherence to the Mediterranean diet, weight reduction, and exercise interventions are related to decreases in markers of systemic inflammation, such as IL-6, TNF-α, and CRP [[45], [46], [47]]. Likewise, the study by Sureda et al. revealed that poor adherence to the Mediterranean diet is directly associated with more adverse plasma inflammation markers, especially among adult males [48]. revealed that poor adherence to the Mediterranean diet is directly associated with more adverse plasma inflammation markers, especially among adult males [48].

Although plaque biofilm is the initial cause of periodontitis, tissue destruction is mainly due to an abnormal inflammatory immune response in susceptible individuals. This hyperinflammatory response is ineffective at eliminating the causative pathogens, leading to the constant release of neutrophil proteolytic enzymes, proinflammatory mediators, and reactive oxygen species (ROS). These factors contribute to the destruction of the periodontal attachment [49]. Previous studies showed proper nutrition is essential to prevent periodontal disease [10]. Nutritional and dietary interventions enhance periodontal therapy outcomes and increasingly become essential tools to regulate host immunity and prevent periodontitis [50].

The findings of the present meta-analysis were in agreement with the study of 1075 young Moroccans, in which no significant association was found between the Mediterranean diet scores (MDS) and periodontal disease [12]. In contrast, the study by Marruganti et al. [3] showed that individuals having a low adherence to the Mediterranean diet are over nine times more susceptible to experiencing severe forms of periodontitis, regardless of their physical activity level. A reason for this inconsistency could be the varied demographics of the populations evaluated in these studies: the former study included university students with an average age of 20 years, whereas the participants in Marruganti et al.'s research were recruited from a public university hospital's periodontal department in Italy, with an average age of 53 years. The college students enrolled by Iwasaki et al. [12] possibly had superior oral health behaviors and were less prone to diseases. Therefore, the two studies have a significant disparity in the reported data regarding the prevalence of periodontitis (6.6 % versus 85 %). In addition, the regression analysis conducted by Radic et al. [41] showed that the MDSS and adherence to daily olive oil consumption following the Mediterranean diet were linked to an increased number of teeth in KTRs. They found no notable relationship between the MDSS score and other parameters related to periodontal disease. Similarly, a recent cohort study examining the relationship between specific dietary patterns and the incidence of periodontal disease found that low adherence to the Mediterranean diet was associated with higher PI and a greater incidence of severe periodontal disease [13]. Additionally, according to Laiola et al., adherence to the Mediterranean diet over eight weeks reduced periodontal disease-related pathogens in obese and overweight subjects [23].

The study by Bartha et al. [19] in 2021 focused on the impact of the Mediterranean diet on gingivitis and showed a beneficial reduction in periodontal inflammatory parameters. Similarly, the study by Woelber et al. [22] in 2019 indicated a comparable but more pronounced decrease in inflammatory parameters, suggesting a potential benefit in opting for a plant-based whole-food diet. Nevertheless, long-term adherence must be further evaluated. Shakeel et al. [42] found a statistically significant decrease in PISA, BOP, BW, and BMI. In the Woelber et al. study [21], the inter-group comparison also reduced probing depth and GI. In contrast, the findings of Bartha et al. [19] indicated no significant disparity for these two parameters. Similar changes in GI for both groups in the Bartha et al. study can be explained by the fact that the PI for the control group decreased from 1.49 to 1.39 during the 8-week study period, while no changes occurred in PI numbers of the Mediterranean diet group. This could have led to similar reductions in GI for both groups. The decrease in the PI in the control group could be explained by the Hawthorne effect – an increase in participants' activity within a study – potentially leading to a subconscious increase in dental hygiene activities of the control group. Conversely, Mediterranean diet group participants focused more on dietary changes than hygiene procedures. Woelber et al. [22] described this effect for both groups, which could indicate that the observed clinical results primarily originate from the change in diet.

Another finding of Woelber et al. [22] was a reduction in omega-6/omega-3 ratio – precursors of pro-and anti-inflammatory lipid mediators – mean value from 8.34 (±1.61) to 7.90 (±1.84). Their study specifically noted arachidonic acid (AA) as the sole omega-6 polyunsaturated fatty acid analyzed. These results were comparable with those of Bartha et al., in 2022, which revealed a mean decrease of around 25 mg/L in AA levels [51].

Ravello et al. [52] reported that for every point gained on the MDI, there are 18 %, 12 %, and 24 % higher likelihood of self-reporting very good/good gingival health, lack of bleeding on toothbrushing, and absence of clinical gingival inflammatory signs, respectively. This study, although relying solely on online self-reported data of periodontal health and dietary habits, had findings that supported those of other studies implementing various methodologies, all of which identified a correlation between adherence to the Mediterranean Diet and periodontal health [21,53].

This review implements a comprehensive and systematic approach and possesses potential strengths and limitations. One of the strengths of this study is the large sample size of the three cross-sectional articles incorporated in the meta-analysis. Regarding the limitations, the number of long-term original studies regarding Mediterranean dietary patterns and periodontal disease has been notably sparse. Moreover, inconsistent heterogenous results have been reported from the three cross-sectional studies included in the meta-analysis [12,13,40], which compromises the statistical significance of our meta-analysis.

## 5 Conclusion

The current study demonstrates no significant association between Mediterranean diet adherence and periodontitis. However, several studies indicated positive effects of the Mediterranean diet on periodontitis. This discrepancy highlights the need for further clinical trial studies with proper design and a larger sample size to better reveal the causal relationship between adherence to the Mediterranean diet and periodontitis. Furthermore, observational studies with an extended follow-up duration need to be conducted.

## Funding

This research received no specific grant from funding agencies in the public, commercial, or not-for-profit sectors.

## Data availability statement

The data supporting this study's findings are available upon request. Interested researchers can contact the corresponding author to obtain access to the data.

## CRediT authorship contribution statement

**Yasmina Aalizadeh:** Writing – review & editing, Investigation. **Nima Khamisi:** Writing – review & editing, Investigation, Conceptualization. **Parastoo Asghari:** Writing – original draft, Validation. **Amirhossein Safari:** Writing – original draft, Investigation. **Mahtab Mottaghi:** Writing – review & editing, Investigation. **Mohamad Hosein Taherkhani:** Investigation. **Anahita Alemi:** Visualization. **Masoume Ghaderi:** Software, Investigation. **Mohammad Rahmanian:** Supervision, Methodology, Formal analysis, Conceptualization.

## Declaration of competing interest

The authors declare that they have no known competing financial interests or personal relationships that could have appeared to influence the work reported in this paper.
